# Preconditioning and Engineering Strategies for Improving the Efficacy of Mesenchymal Stem Cell-Derived Exosomes in Cell-Free Therapy

**DOI:** 10.1155/2022/1779346

**Published:** 2022-05-14

**Authors:** Shenyuan Chen, Fengtian Sun, Hui Qian, Wenrong Xu, Jiajia Jiang

**Affiliations:** ^1^Aoyang Institute of Cancer, Affiliated Aoyang Hospital of Jiangsu University, 279 Jingang Road, Suzhou, 215600 Jiangsu, China; ^2^Zhenjiang Key Laboratory of High Technology Research on EVs Foundation and Transformation Application, School of Medicine, Jiangsu University, 301 Xuefu Road, Zhenjiang, Jiangsu, China

## Abstract

Mesenchymal stem cells (MSCs) have been widely applied to regenerative medicine owing to their multiple differentiation, self-renewal, and immunomodulatory abilities. Exosomes are cell-secreted natural nanovesicles and thought to be mediators of intercellular communication and material transport. The therapeutic potential of MSCs can be largely attributed to MSC-derived exosomes (MSC-exosomes). Emerging evidence suggests that the therapeutic efficacy of MSC-exosomes is highly dependent on the status of MSCs, and optimization of the extracellular environment affects the exosomal content. Pretreatment methods including three-dimensional cultures, hypoxia, and other biochemical cues have been shown to potentially enhance the biological activity of MSC-exosomes while maintaining or enhancing their production. On the other hand, engineering means to enhance the desired function of MSC-exosomes has been rapidly gaining attention. In particular, biologically active molecule encapsulation and membrane modification can alter or enhance biological functions and targeting of MSC-exosomes. In this review, we summarize two possible strategies to improve the therapeutic activity of MSC-exosomes: preconditioning approaches and engineering exosomes. We also explore the underlying mechanisms of different strategies and discuss their advantages and limitations of the upcoming clinical applications.

## 1. Introduction

Mesenchymal stem/stromal cells (MSCs) are multipotent cells capable of high proliferation and self-renewal; in culture, they can differentiate into various cell types such as osteoblasts, chondrocytes, adipocytes, and myocytes [1]. They were first isolated from bone marrow and have since been obtained from many other tissues, including adipose tissue, the Wharton jelly of the umbilical cord, or umbilical cord blood [2, 3]. Owing to their immunomodulatory and tissue regeneration properties, MSCs have been explored for cell therapy [4–6]. However, this ability of MSCs may be mediated by paracrine action rather than differentiation potential [7]. Growing evidence suggests that MSCs release extracellular vesicles (EVs), particularly exosomes, which exert bioactivity similar to that of MSCs [8, 9]. For example, MSC-exosomes can mediate immunomodulation and regenerative effects in various diseases in animal models, including cardiovascular diseases [7], liver diseases [10], diabetes [11], acute lung injury [12], chronic kidney disease [13], Alzheimer's disease [14], stroke [15], and cancer [16]. Apart from their intrinsic biological functions, MSC-exosomes have recently been introduced as promising drug carriers owing to their remarkable biocompatibility, small size, and aptitude for loading specific and diverse therapeutic molecules, including nucleic acids, proteins, and small molecules [17].

Despite the vast potential of natural exosomes, there are still many inherent limitations that restrict their large-scale applications [18]. These limitations are as follows: (A) low yield of exosomes obtained from MSC-conditioned medium under conventional culture conditions [19]. (B) After multiple generations of cultures *in vitro*, the replicative senescence of MSCs usually leads to a decrease in the therapeutic effect of their secreted exosomes *in vivo* [20]. (C) Owing to the inherent properties of natural exosomes, MSC-exosomes are poorly targeted to the site of injury *in vivo* [21]. Therefore, improving the yield of MSC-exosomes and enhancing their disease-specific therapeutic potential or targeting ability is imperative for their clinical applications [18, 22]. The therapeutic potential of MSC-exosomes is closely related to their cellular state [23]. Recent studies have shown that altering MSC cell culture conditions or pretreatment, including three-dimensional (3D culture) [24] and hypoxic culture [25], are adaptive strategies to improve the yield and therapeutic efficacy of MSC-exosomes [18]. On the other hand, engineering exosome approaches can manipulate MSC cells and MSC-exosomes by therapeutic molecular loading or membrane modification to achieve the expected therapeutic goals [26]

In this review, we first summarize the increased therapeutic efficacy of exosomes secreted by MSCs under different preconditioning environments. These pretreatments include 3D and hypoxic environments, cytokines, and other chemical-physical cues [18]. Moreover, we discuss the changes in the contents of these exosomes and the underlying mechanisms of their bioefficacy. Next, we review the available strategies for engineering MSC-exosomes and broadly divide them into two main approaches: endosomal therapeutic molecule piggybacking and membrane surface modification. Molecular piggybacking approaches include endogenous and exogenous cargo piggybacking [17]. Membrane surface modification approaches include genetic manipulation of parental cells, chemical modification, and physical modification, also known as membrane fusion [27]. Finally, we review the applications of various methods in different disease models, while discussing their significance in future development of MSC-exosome therapy.

## 2. Exosomes and MSC-Derived Exosomes

All cells, prokaryotes and eukaryotes, are capable of secreting various types of EVs in a constitutive or inducible manner [17]. As natural nanoscale membrane vesicles encapsulated by phospholipid bilayers, EVs generally fall into three major categories, apoptotic bodies, microvesicles, and exosomes. Apoptotic bodies are the largest subpopulation of EVs and are released by cells undergoing programmed cell death. Microvesicles are vesicles that arise from the surface of the plasma membrane via outward budding [28]. Exosomes, with an average diameter of 30~150 nm, originate through the endocytic pathway [29].

The formation of exosomes is a complex process that includes endocytosis, formation of endosomes and intraluminal vesicles (ILVs), and release of exosomes [30]. First, early endosomes arise from lipid raft domains of the plasma membrane *via* the endocytosis pathway. Then, early endosomes mature into intracellular multivesicular bodies (MVBs) or late endosomes. During this process, ILVs are formed and accumulated in MVBs with the assistance of the Golgi complex. Recent studies have indicated that endosomal sorting complexes required for transport (ESCRT) mechanisms are involved in the formation of MVBs and ILVs [31]. However, the underlying mechanisms for the sorting of cargo into the MVBs and ILVs have not yet been fairly understood [30]. Finally, exosomes are formed after the ILVs contained in MVBs fuse with the plasma membrane and are released into the extracellular environment. When exosomes are released into the extracellular space, they can be internalized into recipient cells by three potential mechanisms: endocytosis, direct fusion with the plasma membrane, and receptor-ligand interaction [32].

**Figure 1:**
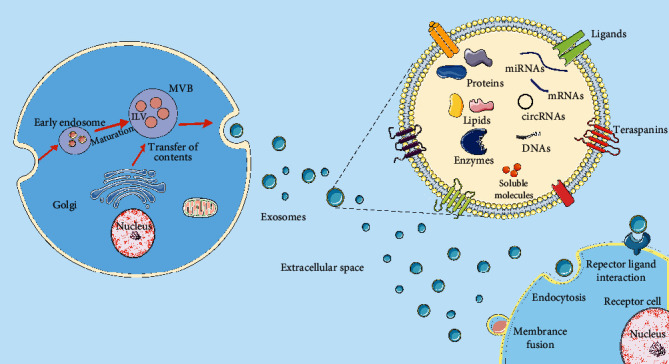
Exosome biogenesis and uptake. Exosomes are generated by the endosomal pathway and released to the extracellular space. Cellular contents, such as proteins, lipids, metabolites, small molecules, DNA, and RNA, along with cell surface proteins can enter exosomes through endocytosis and plasma membrane invagination. Exosomes released by a donor cell can elicit biological responses in a receptor cell by interacting with cell surface proteins or receptors or after being internalized by endocytosis or through membrane fusion.

Depending on the specific physiological or pathological state of the parental cells, exosomes may contain many different components, including DNA, RNA, lipids, proteins, and metabolites [31]. They not only reflect the metabolic state and function of the parental cells but also reach the recipient cells and exchange molecular information [28]. Exosomes derived from MSCs, like other cellular derived exosomes, can carry complex cargo (Figure 1). Currently available data indicate that exosomes can prevent some drawbacks of stem cell therapy (safety and ethical issues), and they have the advantages of high stability, easy storage, no need for proliferation, and ease of quantitative use [33]. Importantly, MSC-exosomes have greater tissue regenerative potential as they contain multiple proteins and RNAs, compared to a single cytokine [34]. To date, hundreds of miRNAs enriched with MSC-exosomes have been identified based on RNA sequencing. They can regulate gene expression in recipient cells through degradation and reexpression and play important role in angiogenesis [35], tissue recovery [36], immune regulation [37], and neuroprotection [38]. Moreover, proteins are considered to be the main regulators of tissue repair and regeneration in MSC-exosomes [39], including Wnt4 [40], 14-3-3*ζ* [41–43], GPX1 [44], casein kinase 1*δ* (CK1*δ*) [45], *β*-TRCP [45], growth factors, and angiogenic factors [46].

Notably, the generated exosomes do not constitute a uniform population. Their composition may vary depending on many factors, such as the culture environment [47], state of the MSC [48], the isolation procedure, and their cellular origin [49]. MSC-exosomes are easily captured by the reticuloendothelial system, including the liver, spleen, and other immune organs [50], and it has been shown that the targeting ability of MSC-exosomes remain inefficient in vivo animal experiments [27]. It may prevent MSC-exosomes from exerting therapeutic efficacy at the site of injury. There is an urgent need to develop methods to improve the yield, targeting, and ultimately, the efficacy of these exosomes without compromising their function.

## 3. Extracellular Environment Preconditioning

### 3.1. 3D Culture Environment

Conventionally, MSCs are generated through the conventional static adherent cultures, known as 2D culture, in the presence of fetal bovine serum (FBS) or human-sourced supplements. To the best of our knowledge, there are differences between 2D culture environment and 3D microenvironment in vivo, such as biological activity, medium structure, and nutrient release. In a 2D environment, MSCs may gradually lose their original traits, morphology, structure, and function, result into the worse proliferation and differentiation of MSCs [51, 52], which would impact the therapeutic properties of exosomes. Therefore, the production efficiency of exosomes under conventional culture is often extremely low [52, 53]. 2D culture may not meet the anticipated demand for stable quality-assured MSCs in future cell-free therapies.

At present, 3D culture technology can be broadly divided into two types: material-free cultures (cell spheres formed by aggregation of cells) and material-supported cultures. Material-free 3D culture methods mainly include 3D spherical spatial boundary conditions [54], scaffold-free suspension cultures [55], systematic microcarrier screening, hydrogel-assisted 3D culture [56, 57], and agitated culture conditions [58]. Material-supported culture methods are relatively diverse, including fibrous scaffolds [59], native extracellular matrix (ECM) bioscaffolds [60], hollow fiber bioreactors [61], quantum cell expansion systems [62], and computer-controlled bioreactors [63]. These approaches allow cells to establish a tight connection with the culture environment, forming a 3D structure through dynamic interactions. Owing to their suitable void structure, surface activity, mechanical strength, and biocompatibility, 3D culture systems can produce large numbers of MSCs [52, 54, 58] and MSC-exosomes [57, 58, 61, 64] in a shorter time or within a small volume. 3D culture systems based on hollow fiber bioreactors increase the total MSC-exosome production about 19.4 times than 2D culture [61]. Meanwhile, MSCs usually exhibit better differentiation efficiency in 3D culture systems than in conventional cultures [60].

At present, 3D-exosomes have unique advantages in diverse injury repair, such as endothelial cell proliferation, migration, and angiogenesis [24]. 3D-exosomes could alleviated cisplatin-induced acute kidney injury more significantly as well [64]. Besides, 3D-exosomes have shown promising application prospects in the treatment of central nervous system diseases [63, 65, 66]. For example, 3D-exosomes collected in 3D collagen scaffolds could significantly improve functional recovery and neurovascular remodeling in rats after traumatic brain injury. Teixeira et al. found that the implementation of computer-controlled suspension bioreactors could enhance the neuroregulatory spectrum in the MSC-secreted group and increases the secretion of some neuroregulatory molecules and miRNAs. After injection of dynamically cultured MSC-exosomes, the survival and differentiation of neurons and astrocytes in the hippocampal dentate gyrus (DG) of the rat brain were enhanced [63]. 3D-exosomes could improve Alzheimer's disease-like phenotypes in preclinical mouse models as well [66]. Some emerging microfluidic-based tools in 3D cell cultures have also gained interest. These combinatorial systems have developed novel therapeutic strategies based on EVs of MSC origin to naturally overcome bacterial defense mechanisms [67].

### 3.2. Hypoxic Preconditioning

Generally, oxygen tension is considered as one of important factors that affects the biological behavior of stem cells during culture [68–70]. Physiological oxygen tension varies in different tissues and is approximately 12% in blood and as low as 1% in deep cartilage tissue [71]. In the bone marrow, oxygen tension is between 4% and 7% or as low as 1–2%. It is widely believed that the oxygen tension of cells in the body is lower than that in the atmosphere (21%) [71, 72]. As early as 1958, Cooper et al. found that when cells were cultured under conditions of lower oxygen, the proliferation ability of many specific cell types was stronger [73], MSCs as well [74], while the atmospheric oxygen concentration may cause cellular DNA damage and lead to genetic instability [70].

Studies have indicated that a low-oxygen tension (hypoxia) may improve the proliferation and genetic stability of MSCs by activating glycolysis [75]. At the same time, hypoxia promotes differentiation potentials and upregulates stemness genes, including Oct4, Nanog, and Sox2 [69, 76]. In addition, reduction in the oxygen tension is expected to lead to activation of the hypoxia-inducible factor (HIF-1*α*), which activates the transcription of angiogenic genes such as vascular endothelial growth factor (VEGF) and C-X-C motif chemokine receptor 4 (CXCR4), [77, 78]. VEGF and CXCR4 could finally increase the paracrine and migratory capacity of MSCs [77]. Hypoxic incubation may provide a better state of MSCs and incur undoubtedly better therapeutic efficacy in some respects.

The mechanisms underlying the beneficial effects of hypoxic tension on MSCs and exosomes secreted by MSCs cultured in hypoxic environments (Hyp-MSC-Exos) have been gaining much attention in recent years [79]. In fact, a growing number of studies have found that hypoxic stimulation enhances the therapeutic potential of MSC-exosomes for a variety of diseases, including insufficient vessel growth [80, 81], myocardial infarction [25, 82–84], spinal cord injury [85], fracture [86], and diabetic wound [47]. Hyp-MSC-Exos can induce the expression of HIF-1a in human umbilical vein endothelial cells (HUVECs) and promote angiogenesis *in vitro*, by carrying Jagged-1 [80, 87] and high mobility group box 1 protein (HMGB1) [81]. Jagged-1 in Hyp-MSC-Exos, as a ligand for notch, can induce expansion of haematopoietic stem cells through stimulating the Notch signaling pathway, which in turn alleviates bone marrow transplantation [87]. Recently, based on high-throughput sequencing, a series of specific miRNAs enriched with Hyp-MSC-Exos, including miR-486-5p [25], miR-125b [82], miR-216a-5p [85], miR126 [86], miR-126-3p [88], miR-21-3p [47], and miR-31-5p [47], are potentially involved in mediating the treatment of diseases. Liu et al. found that miR-216a-5p, most enriched in Hyp-MSC-Exos, may mediate TLR4/NF-*κ*B/PI3K/AKT signaling pathway to regulate microglial polarization and thereby alleviate spinal cord injury [85]. Another study found that Hyp-MSC-Exos may inhibit inflammation and promote diabetic wound healing by targeting the PI3K/AKT signaling pathway [47]. Compared to normoxic MSC-exosomes, Hyp-MSC-Exos are more easily taken up by cells [85]. This phenomenon indicates an opportunity for hypoxia treatment to improve exosome utilization indirectly.

However, from bone marrow MSC-EVs of five healthy donors, there are limited effect of hypoxia on the miRNA landscape in MSC-EVs and high interindividual variation between hypoxic and normal MSC-EVs [89]. It is not clear whether these inconsistent results are related to the cells from different donor variabilities between individuals or the extraction method of exosomes. Thus, we suggest that this issue should be researched further by using the same panel of cell lines and the same number of cells in each batch of a project.

### 3.3. Cytokine Preconditioning

The paracrine efficiency of MSCs and the therapeutic activity of their exosomes are spurred by physiological needs [90]. Cytokine [74] and inflammatory stimulation [90] have been proved to improve paracrine efficiency and regulate the production and excretion of different potential therapeutic factors in MSC, including exosomes.

One of the most studied cytokines that trigger the therapeutic potential of MSC-exosomes is tumor necrosis factor-alpha (TNF-*α*) [91–94]. Exposure to TNF-*α* enhances the therapeutic efficacy of MSC-exosomes for several diseases, involving periodontitis [91], acute liver failure [92], urethral stricture [94], and retinal ganglion injury [93]. Mechanistically, induction by mild TNF-*α* shifts MSCs to a more anti-inflammatory phenotype, which results in the release of more anti-inflammatory exosomes. It was observed that inflammatory suppression-related miRNAs, including miRNA-299-3p and miR-146a, was enriched in those exosomes [92, 94].

Another inflammatory mediator known to induce the regenerative capacity of MSC-exosomes is interleukin-1*β* (IL-1*β*). A recent study found that an IL-1*β* treatment increased the anti-inflammatory effects of MSC-exosomes in astrocytes mediated through the NRF2 signaling pathway [95]. Additionally, it was suggested that IL-1*β*-pretreated MSC-exosomes had better anti-inflammatory effects in both sepsis and osteoarthritis model [96–98]. In addition to inflammatory cytokines, Yang et al. demonstrated that IFN-*γ* also enhances the ability of MSC-exosomes to inhibit Th17 cell differentiation and reduce colitis [99]. Similar to TNF-*α*, IL-1*β* alters the secretory profile of MSC and upregulates the levels of miR-146a [96], miR-21 [97], and miR-147b [98] in exosomes, while IFN-*γ* pretreatment can increase miR-125a and miR-125b levels [99]. These miRNAs effectively support the healing and repair in specific diseases. Collectively, these data suggest that inflammatory stimuli enhance the regenerative potential and anti-inflammatory response of MSC-exosomes.

### 3.4. Other Chemical and Physical Preconditioning

MSCs are responded rapidly to environmental stimuli [74]. Except the 3D environment, hypoxic condition, and inflammatory factors, MSCs can be stimulated by a variety of different chemical and physical signals that may significantly alter their phenotype. And these changes also affect the amount and content of exosomal secretion, which have broad applications in tissue regeneration and immune modulation [100]. For instance, it was recently shown that metformin pretreatment promoted the release of MSC-exosomes via an autophagy-associated way. Besides, metformin mediated the transfer of ITIHT4 (interalpha-trypsin inhibitor heavy chain H4) into MVBs, resulting in the accumulation of ITIH4 in the released MSC-exosomes. These processed MSC-exosomes could ameliorate nucleus pulposus cells senescence *in vitro* and optimize the therapeutic potential in IDD (intervertebral disc degeneration) [101]. Another study found that MSCs exposed to blue (455 nm) monochromatic light released exosomes [37] with increased levels of miR-135b-5p and miR-499a-3p. Blue light irradiation also enhanced the proangiogenic capacity of MSC-exosomes *in vitro* and *in vivo*, thereby improving their therapeutic efficacy [102].

Treatment with other chemically factors such as EP4-antagonist [103], glycyrrhetinic acid (GA) [104], kartogenin [105], thrombin [106], oridonin [107], heme oxygenase-1 (HO-1) [108], atorvastatin (ATV) [109, 110], and melatonin [111–114] as well as other physical induction methods such as titanium surfaces [115], low-intensity pulsed ultrasound (LIPUS) [116], PG/TCP (PEGMC with *β*-TCP) [117], extrusion [118], and bioglass [119] could improve the efficacy of MSC-exosomes therapy. These methods are relatively inexpensive and rarely require special instrumentation and appear to be more worthwhile as clinical strategies. In Table 1, we provide a brief review of these methods with the hope that they will inspire future research directions.

**Figure 2:**
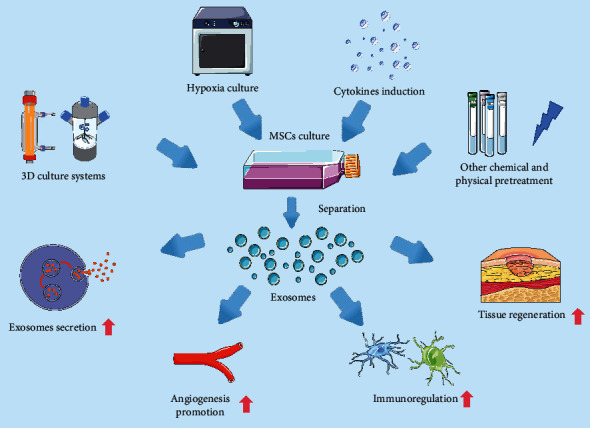
Current preconditioning patterns used to increase the therapeutic efficacy of MSC-exosomes. A 3D culture was used to increase the secretion of MSC-exosomes and enhance their tissue damage repair efficacy. A hypoxic environment may promote MSCs to secrete more exosomes; exosomes of hypoxic MSC origin perform better in proangiogenesis, immunomodulation, and tissue repair. Pretreatment with different cytokines can effectively enhance the therapeutic potential of MSC-exosomes, particularly their immunomodulatory ability and tissue damage repair efficacy. Other chemical and physical stimuli may alter the state and exosomal content of MSCs, thus enhancing their therapeutic potential in certain disease models.

Together, these data suggest that different physical or chemical cues can enhance the regenerative or anti-inflammatory potential of MSC-exosomes by modulating the secretory profile of MSCs (Figure 2). However, due to the heterogeneity of MSCs from different sources, the appropriate intensity and time of pretreatments still need to be further explored by researchers.

## 4. Engineering MSC-Derived Exosomes for Improving Therapeutic Potential

**Figure 3:**
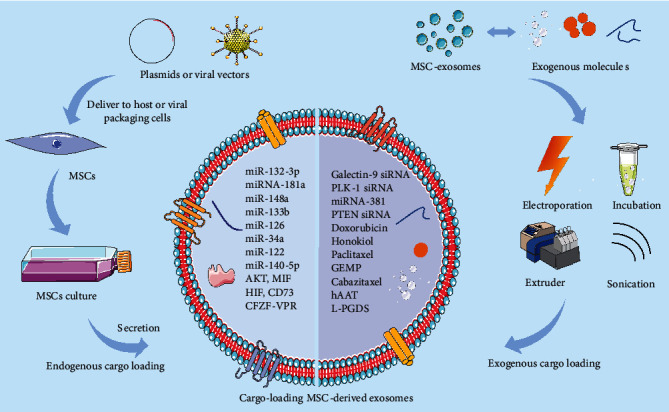
Cargo-loading approaches to enhance the therapeutic potential of MSC-exosomes. Endogenous cargo piggybacking is the use of plasmids or viruses to construct genetically modified parental MSC cells, followed by the collection of endogenous cargo-packed exosomes from cell culture supernatants. Endogenous cargoes are usually miRNAs and proteins with therapeutic benefits. An exogenous cargo is piggybacked by the delivery of drugs or therapeutic small molecules into exosomes using methods such as electroporation, coincubation, or ultrasound.

In the last decade, advanced studies have led to the emergence of a new field of EV engineering [17, 20]. At the same time, these approaches have been used to modify MSC-exosomes in order to enhance their therapeutic potential [120]. Exosome engineering aims to achieve the following goals: (A) loading endogenous or exogenous molecules (nucleic acids, proteins, or drugs) into the lumen of exosomes (Figure 3); (B) surface modification of MSC-exosomes in order to target exosomes to a particular type of tissue or cells [121]. In this section, we review techniques used to engineer MSC-exosomes and discuss their application in various disease models.

### 4.1. Cargo Packaging into Exosomes

#### 4.1.1. Endogenous Cargo Loading

Previous studies have suggested that functional molecules in MSC-exosomes, such as proteins and nucleotides, are involved in immune regulation, tissue regeneration, and angiogenesis in various disease models [33]. By using biological tools (plasmids or viral vectors), MSCs can be genetically manipulated to promote the expression of endogenous molecules, which can be encapsulated inside MSC-exosomes by utilizing the cell's biomolecular synthesis mechanism. Therefore, the strategy of loading exosomes with therapeutic molecules through genetic engineering based on parental cells may improve the therapeutic efficacy of MSC-exosomes and prevent the heterogeneity between different batches of exosomes.

Accumulating evidence show that MSC-exosomes mediate the therapeutic effects of parental cells in various disease models through miRNA delivery. Pan et al. used lentivirus to transfect miR-132-3p mimics, an angiogenic miRNA, into MSCs, and successfully isolated exosomes overexpressing miR132-3p. *In vitro*, compared with natural exosomes, miR132-3p-overexpressing exosomes could deliver miR-132-3p to the target cells, which could reduce the generation of ROS, apoptosis, and interruption of tight junctions in the EC of H/R damage more effectively [122]. Similarly, genetically modified MSC-exosomes overexpressing functional miRNAs (miRNA-181a or miR-148a) exert stronger therapeutic effects on myocardial ischemia perfusion injury [123] and liver ischemia-reperfusion injury [124], respectively.

Exosomal miRNAs also play as key regulators in the pathogenesis of various neurological diseases [125]. Shen [126] et al. found that miR-133b-modified MSC-exosomes in SD rats with cerebral hemorrhage mediated the neuroprotective effect of RhoA and ERK1/2/CREB against apoptosis. Besides protecting neurons, MiR-133b-modified MSC-exosomes also mediate the transfer of miR-133b to astrocytes, thereby regulating gene expression and promoting neurite remodeling and functional recovery after stroke [127]. In addition, MSC-exosomes modified by miR-126 and miR-26a protect neurons in rats with spinal cord injury, stimulate axon regeneration, and improve the recovery of limb motor function after spinal cord injury [128, 129].

Fibrosis can occur in many organs, including the kidneys and liver, which seriously endangers human life and health [130, 131]. It has been reported that miR-34a exhibits direct antifibrotic effects on hypoxia-induced fibrosis in human kidney-2 (HK-2) renal tubular cells. He et al. transfected MSCs with miR-34a-overexpressing lentivirus, collected miR-34a-overexpressed MSC-exosomes, and evaluated its effects on transforming growth factor *β*- (TGF-*β*-) induced fibrosis and apoptosis [132]. The study showed that miR-34a overexpression of MSC-exosomes can inhibit the EMT of renal tubular epithelial cells, possibly by inhibiting the Jagged-1/Notch-1 pathway. Lou et al. found that miR-122 (which negatively regulates collagen production in HSCs) modification through exosomal-mediated miR-122 communication improves the efficacy of MSCs in the treatment of carbon tetrachloride- (CCl4-) induced liver fibrosis [131].

In addition to miRNAs, the piggybacking of endogenous proteins (enzymes [133–135], cytokines [136], and transcription factors [137]) has improved the therapeutic efficacy of MSC-exosomes in a variety of ischemic diseases. Wei et al. used an adenoviral transfection system to transfect human antiprotease alpha-1 antitrypsin (hAAT) into MSCs (hAAT-MSCs) and found that hAAT-MSCs -exosomes exhibited more beneficial effects in immunoregulation [134]. Similarly, exosomes secreted by bone MSCs overexpressing MIF (a cytokine that plays a key role in regulating cellular homeostasis) had better cardioprotective effects in a rat model of myocardial infarction [136]. In addition, related studies have identified the beneficial regulatory roles of HIF in angiogenesis. The overexpression of HIF in MSCs-exosomes exerted a better proangiogenic effect in cranial defects [138], myocardial infarction [139], and early ischemic necrosis of the femoral head [140]. A recent study found that CD73-modified MSC-derived EVs could break down overreleased adenosine triphosphate (ATP) and improve the M1 to M2 polarization phenotype of microglia, thereby improving locomotion in mice with spinal cord contusion [141].

Cystic fibrosis (CF) is an autosomal recessive disorder caused by mutations in the CFTR gene. Therapies to enhance or correct the CFTR have shown clinical benefits. Villamizar et al. developed an MSC-exosome equipped with a zinc finger activator protein (a transcriptional activation complex targeting the CFTR promoter) based on genetic reprogramming techniques. These exosomes were able to target the promoter (CFZF-VPR) of CFTR in human bronchial epithelial cells (HuBEC) and activate transcription [137]. This new approach provides a novel next-generation gene therapy based on MSC-exosomes for the treatment of patients with CF.

#### 4.1.2. Exogenous Cargo Loading

MSC-exosomes loaded with endogenous cargo enhance the natural repair process by mimicking their natural biological functions. On the other hand, owing to their inherent low immunogenicity and enhanced permeation retention (EFR) effects, recent studies have revealed that exogenous therapeutic entities delivered by MSC-exosomes could be used as natural drug carriers with great potential in cancer therapy [26].

At present, electroporation is the most common method for loading exogenous “cargo” into EVs [142]. The principle of this method is to utilize an external electric field to generate small repairable pores in the phospholipid bilayer, allowing various small molecules into EVs under an electric field force to realize drug loading. Anticancer drugs, such as doxorubicin, can be delivered efficiently into MSC-exosomes by electroporation and can reduce significantly tumor growth rates in mouse models of colon [143] and breast cancer [144]. Liang et al. successfully piggybacked the exogenous anticancer drug norcantharidin (NCTD) into MSC-exosomes using electroporation. These engineered exosomes were used for the treatment of mouse liver cancer models owing to the natural liver-targeting properties of exosomes [145].

Small interfering RNAs (siRNAs) and miRNAs have outstanding advantages and great potential for application in targeted cancer therapy. Specific targeting of siRNA or miRNA drugs to disease-causing genes can lead to more precise and personalized treatment [146]. Unfortunately, there are still significant challenges with systemic administration of RNA drugs, such as how to obtain a siRNA or miRNA vector with long survival time *in vivo*? In recent years, as natural drug carriers, MSC-exosomes loaded with specific exogenous siRNAs or miRNAs have also been used for cancer therapy. For example, in 2021, a study used MSC-exosomes loaded with galectin-9 siRNA to inhibit the polarization of macrophage proliferation and downregulate Tregs to induce antitumor immunity. Galectin-9 siRNA has achieved remarkable results in the treatment of pancreatic ductal adenocarcinoma (PDAC) [147] models. Greco et al. wrapped polo-like kinase 1PLK-1 siRNA into MSC-exosomes by electroporation and delivered them to silence PLK-1 in bladder cancer cells [148]. According as that miRNA-381 is aberrantly expressed in triple-negative breast cancer (TNBC) and restoring its expression could limit the invasiveness of TNBC, Shojaei et al. utilized electroporation to package miRNA-381-3p mimic into MSC-exosomes and delivered it to TNBC cells, thereby inhibiting their migration ability [149].

Moreover, coincubation [150], sonication [151], and dialysis [152] have been used to piggyback exogenous molecules to MSC-exosomes. For instance, researchers loaded the siRNA of phosphatase and tensin homologs (PTEN) into MSC-exosomes by coincubation (ExoPTEN) to enhance the clinical therapeutic and application potential of MSC-exosomes for spinal cord injury. When administered intranasally, ExoPTEN can migrate across the blood-brain barrier (BBB) into the spinal cord. *In vivo*, these exosomes greatly enhanced axonal regeneration and restored motor function in SCI rats [150].

In fact, the strategy of combining multiple exogenous mounting methods to mount multiple anticancer drugs in MSC exosomes has been continuously explored. For example, to overcome chemoresistance in pancreatic ductal carcinoma (PDAC), Zhou et al. used both electroporation and ultrasound techniques to load paclitaxel (PTX) and gemcitabine (Gem), respectively, into MSC-exosomes [153]. Those engineered exosomes showed promising antitumor benefits *in vitro* and *in vivo* owing to the synergistic effect of PTX and GEM and the penetrating properties of the exosomes. In cancer therapy, engineering MSC-exosomes are already under clinical assessment for future use in nanoscale drug delivery [154]. For example, a phase I clinical trial (NCT03608631) will investigate the dose, effects and safety of MSC-derived exosomes with KrasG12D siRNA (iExosomes) in the treatment of participants with pancreatic cancer with KrasG12D mutation [155].

The rapid development of nanotechnology has focused attention to combine multiple engineering approaches to modify exosomes. On the one hand, the combined use of exogenous piggybacking of MSCs exosomes and endogenous genetic modifications may provide new insights for the treatment of tumors. For example, Qiu et al. coincubated a drug (cabazitaxel, CTX) for the treatment of oral squamous cell carcinoma with tumor necrosis factor-related apoptosis-inducing ligand (TRAIL) gene-modified MSCs and successfully purified exosomes loaded with CTX/TRAIL (MSCT-EXO/CTX). Subsequently, the anticancer effects of MSCT-EXO/CTX were successfully validated *in vitro* and *in vivo* [156]. On the other hand, the combined modification of therapeutic molecular piggybacking and membrane surface modification of MSC-exosomes has received increasing attention for its potential to improve the targeting of tumor tissues [147, 157]. In the following section, we focus on the literature on membrane surface modification.

### 4.2. Surface Modification of Exosomes

**Figure 4:**
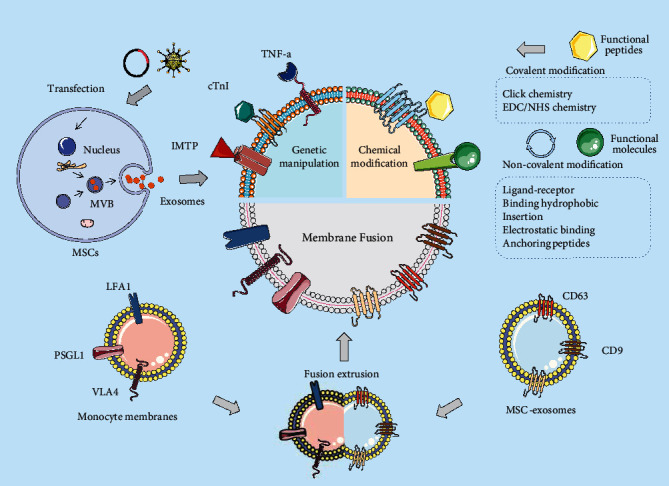
Three common strategies for membrane surface modification of MSC-exosomes include genetic engineering, chemical modification, and membrane fusion. Genetic engineering fuses gene sequences of guidance proteins or peptides with those of selected exosomal membrane proteins and can effectively display specific guidance peptides and proteins on the exosomal surface. In contrast, chemical modifications display a variety of natural and synthetic ligands or receptors on the membrane surface through covalent or noncovalent modifications. Membrane fusion uses extrusion to fuse exosomes with other membrane structures, an approach that confers new functional and therapeutic benefits to them.

Exosomes can penetrate tissues, diffuse into the blood, and cross the BBB. After MSC-exosomes enter the blood circulation, their tissue targeting must be improved for better therapeutic benefits. However, the fusion of exosomes with recipient cells is not random, and some molecules contained in exosomes contribute to targeting specific tissues, while others ensure that the probability of nonspecific interactions is minimized. Although MSC-exosomes may be chemotactic toward regions of inflammation or injury owing to the interaction between SDF-1 and CXCR4, their ability to target specific regions and their active transport in vivo remain insufficient to meet the expected demands [158]. Recent studies have demonstrated that genetic modification techniques based on parental cells such as molecular cloning and lentiviral packaging techniques can produce engineered exosome populations with targeted properties [158]. EVs with unique protein modifications are thought to contain the barcodes needed to find specific target cells. Consequently, researchers have developed specific cell-targeted delivery tools based on engineered exosomes. Cellular and tissue specificity is conferred by the modification of exosome surface molecules. Membrane surface modification strategies include genetic modifications, chemical modifications, and membrane fusion (Figure 4).

#### 4.2.1. Genetic Manipulation

Parental cell-based genetic engineering techniques, such as molecular cloning and lentiviral packaging, can generate engineered exosome populations with targeted properties^151^. Genetic engineering fuses gene sequences of guidance proteins or peptides with those of selected exosomal membrane proteins, which can effectively display specific guidance peptides and proteins on the exosomal surface. First, the ligand or targeting peptide is fused to the gene for the transmembrane protein expressed on the exosomal surface. Secondly, donor cells are transfected with a plasmid encoding the fusion protein, and the donor cells secrete engineered exosomes with the targeting ligand on their surface.

As cTnI is released and maintained at high concentrations in the myocardial infarct region, Wang et al. designed plasmids targeting the cTnI short peptide and used gene transfection in MSCs to obtain cTnI-targeted exosomes. They found that these exosomes could be localized to the infarct zone along the cTnI concentration gradient. They can be efficiently endocytosed by cardiomyocytes, thus promoting cardiomyocyte proliferation around the infarct zone, ultimately restoring the cardiac function [159]. Recombinant transmembrane proteins of exosomes is another common engineering method to modify the surface of exosomes, such as lysosomal-associated membrane protein 2b (Lamp2b), an exosomal surface protein with an exosomal signal peptide. Through genetic engineering, the protein of interest can be fused with Lamp2b on the surface of exosomes as a target. Ligands or homing peptides displayed on the external surface of exosomes increase the specificity and efficiency of delivery [160]. Recently, Wang et al. [121] employed phage display techniques and identified a new peptide sequence, CSTSMLKAC (IMTP), which can preferentially target areas of cardiac ischemia. Subsequently, they genetically modified MSCs using lentiviral packaging technology, and IMTP was fused to the C-terminus of Lamp2b and introduced to the surface of MSC-exosomes (IMTP-exosomes). In a mouse model of myocardial infarction, IMTP-exosomes significantly increased the targeting of cardiac cells and inhibited cardiomyocyte apoptosis.

Other than improving targeting, genetically manipulated exosome membrane modifications can be used to mount therapeutic molecules [161]. A study in 2021 showed construction of a recombinant expression vector for a cell membrane penetrating peptide (CPP) and TNF-*α* (CTNF-*α*) fusion protein and successfully introduced it into MSCs (CTNF-*α*-MSC). Subsequently, membrane TNF-*α*-anchored exosomes were obtained from CTNF-*α*-MSC and proved to be membrane anticancer activity [161]. Tsai et al. constructed two membrane-localized form vectors (FGL1-TM vector and PD-L1 vector) and transferred together into MSCs (FP-MSCs) using a bioengineering strategy [162]. FP-MSCs-derived exosomes not only expressed abundant FGL1/PD-L1 but also maintained the immunomodulatory properties of unmodified MSC-exosomes. These engineered dual-targeted exosomes were demonstrated to have the potential to alleviate transplant rejection.

#### 4.2.2. Chemical Modifications

Chemical modifications display various natural or synthetic ligand receptors on the surface of exosomes through various techniques, which can be broadly classified into covalent and noncovalent modifications.

The surface of EVs is characterized by the presence of amine groups and carboxy-terminated phospholipids, which can form chemical bonds with different functional groups directly and quickly, also called covalent modification. It includes click chemical amide condensation and covalent coupling methods [163]. The most obvious purpose of the covalent modification is to immobilize targeting moieties onto MSC-exosomes; the most commonly used strategy for covalent modification is click chemistry [164]. Classical click chemistry is a cycloaddition reaction between two functional groups, alkyne and azide, catalyzed by copperions. Jia et al. used click chemistry to conjugate the glioma targeting ligand NRP-1 specific ligand (RGE-peptide) to the surface of MSC-exosomes. The modified exosomes could successfully cross the BBB and helps to accurately identify gliomas [165]. In a 2018 study, a more rapid and convenient biogonormal chemobiology method, copper-free azide alkyne cycloaddition, was applied to MSC-exosome surface modification [166]. Specifically, a C (RGDYK) peptide with two end groups of azide groups was coupled to the MSC-exosome surface (CRGD-ExO) *via* a copper-free azide alkyne cycloaddition reaction. Owing to the high affinity of the C (RGDYK) peptide specifically for integrin *α*V*β*3 in postischemic reactive cerebrovascular endothelial cells, CRGD-ExO successfully targeted the region of ischemic brain injury in a transient middle cerebral artery occlusion (MCAO) mouse model after intravenous administration [166].

However, it has been suggested that the one-step direct functionalization of exosomes by click chemistry is an uncontrolled and time-consuming process; in contrast, the formation of amide covalent bonds between carboxyl groups and exosomal amines by 1-ethyl-3-[3-dimethylaminopropyl] carbodiimide hydrochloride/N-hydroxysulfosuccinimide (EDC/NHS) chemistry is a mild covalent modification method [167]. The LJM-3064 aptamer shows a strong affinity for myelin and has been proven to induce myelin regeneration. Accordingly, Hosseini made the carboxylic acid functionalized LJM-3064 aptamer covalently bounding to the amine groups on the surface of exosomes by EDC/NHS chemistry; this aptamer-exosome biological conjugates as a preventive measure is effective in reducing inflammation and neurodegeneration of multiple sclerosis (MS) [168]. In recent years, specific drugs have also been used for surface covalent modification of MSC-exosomes to better exploit the therapeutic potential of MSCs. Oxaliplatin (OXA), a component of the FOLF-IRINOX regimen for PDAC treatment, triggers ICD effects at the pancreatic tumor site and kills tumor cells by inhibiting DNA synthesis and repair [169]. Zhou et al. first oxidized oxaliplatin to OXA (IV) OH and then modified it with N-(2-aminoethyl) maleimide to synthesize a prodrug form (OXA-Mal) covalently conjugated with MSC-exosomes via vortexes to obtain a stable maleimide-mercaptans conjugate and ultimately achieve drug delivery [147].

Besides covalent modification strategies, noncovalent modification techniques, including ligand-receptor binding [161], hydrophobic insertion [170], electrostatic binding [171], and anchoring peptides [172], have also been applied to the modification of exosome membrane surfaces. To overcome the limitations of gene manipulation and click chemistry, Gao et al. used phage display to identify and select a polypeptide CP05 that specifically binds to the second extracellular ring of CD63. The drug PMO for the treatment of Duchenne muscular dystrophy (DMD) was conjugated to CP05 by an amide bond. The systemic injection of exosomes containing CP05-modified PMO produced an 18-fold increase in the quadriceps of antidystrophin-deficient mdx mice [172], compared to natural exosomes.

However, noncovalently bound species are susceptible to dissociation owing to changes in temperature, solution, and ionic strength, which may limit the range of materials and applications for their modification [158]. Therefore, researchers tend to combine several engineering strategies to tackle the multiple membrane barriers during drug delivery in vivo. For example, Huang et al. developed a MSCs-exosome-based self-directed nanovector, called PR-EXO/PP@Cur, for the treatment of Parkinson's disease via intranasal administration [173]. To improve the neural cell targeting of MSCs-exosomes, the octadecyl chains modified with penetratin (SA-P) and rabies virus glycoprotein peptides (SA-RVG29) were embedded into the exosomal membrane through hydrophobic carbon chains (PR-EXO). Then, PR-EXO and PP@Cur (a self-assembly micelle encapsulated curcumin and SPIONs) were constructed PR-EXO/PP@Cur nanocarriers by ultrasound. The combination of multiple engineered technologies allows PR-EXO/PP@Cur to release drugs directly into the cytoplasm of nerve cells after crossing multiple barriers *in vivo*.

#### 4.2.3. Membrane Fusion

In recent years, the cell membrane coating technology has attracted attention in nanomedicine. This method obtains suitable cell membranes by hypotonic treatment, repeated freeze-thawing, or ultrasonic disruption and then coextrudes with nanoparticles through porous polycarbonate membranes. It combines the natural cell membrane properties with those of nanoparticles, thus greatly enhancing the long-lasting circulation and targeted delivery of nanoparticles *in vivo* [21, 174]. In fact, exosome membranes possess a lipid bilayer structure that spontaneously fuses with various lipid membrane structures. Yang et al. have innovatively investigated a new biocompatible technique, called membrane-editing, using virus-mimetic fusogenic exosomes to transfer functional membrane proteins directly into cellular membranes [175]. This delivery technique based on fusogenic exosomes also enables exosomes to target specific tissues. For instance, in a study conducted in 2020, exosomes modified with monocyte membranes (Mon-Exos) were generated by modifying MSCs-exosomes with monocyte mimics using fusion extrusion, to improve the delivery efficiency of exosomes to myocardium with ischemic damage [176]. Mon-Exos were extremely efficient in targeting the injured heart muscle by stimulating the recruitment of monocytes after myocardial infarction/RI, which ultimately improved cardiac function and histopathological changes in the mouse MI/RI model after treatment [176]. Undoubtedly, the modification of MSC-exosomes by membrane fusion opens new possibilities to improve their cell-targeting therapeutic efficiency.

## 5. Conclusions and Perspectives

The therapeutic benefits of MSC-exosomes have been demonstrated in scenarios involving inflammation regulation [177], tissue damage repair [178], and tumor suppression [179]. However, some weakness such as low yield, heterogeneity, and poor targeting have limited their widespread applications [18, 180]. Consequently, recent studies have focused on developing preconditioning and engineering methods to produce a uniform population of targeted MSC-exosomes that exert enhanced therapeutic benefits [181]. 3D and hypoxic culture conditions, as well as cytokine and chemical pretreatment, have been used to maximize the therapeutic viability of MSC-exosomes [25, 66]. These approaches are easy to operate while yielding uniformly enriched therapeutic molecules in MSC-exosomes. Bioengineering techniques (therapeutic molecules loading and surface modification) have been utilized to manipulate certain components, thus increasing the therapeutic efficacy and targeting capabilities of MSC-exosomes [137, 182].

Despite these promising results, there are disadvantages to both preconditioning and engineering methods: (A) pretreatment of MSCs with either chemical or physical signals fail to reduce the nonspecific aggregation of their exosomes during treatment [23]. (B) The long-term effects of biochemical preconditioning (such as cytokines and chemical cues) on the physiological properties of MSCs still need to be evaluated in detail [18]. (C) Engineering techniques fail to consistently obtain the desired exosomes and require additional transfection, physical loading or chemical modification steps, making their industrial adoption relatively difficult [183, 184]. (D) The potential impacts of engineering techniques on protein and nucleic acid drugs need to be carefully considered [185]. (E) The size of the drug during exosomes loading poses another challenge [186].

Accordingly, addressing these limitations may increase the possibility of their applications in translational medicine [187]. Fortunately, to date, many clinical trials have begun to evaluate the influence of various external factors on the therapeutic efficacy and safety of MSC-exosomes [154, 155, 188]. The availability of cost-effective large-scale production, isolation, and characterization methods of exosomes are already being explored [186, 189]. A growing number of new analytical techniques promise to provide new insights into the uniqueness of exosomes and may stimulate the development of the next generation of modified MSC-exosomes [190].

## Figures and Tables

**Table 1:** Chemical and physical pretreatment for improving therapy potential of MSC-exosomes.

Pretreatment	Source	Potency	Molecular mechanism	Ref.
EP4-antagonist	hBMSC	Promoting neurogenesis and neuritogenesis in damaged hippocampi.	Increasing conversion of 2,3-cAMP to adenosine and promoting *β*3-tubulin polymerization *in vitro*.	[96]
Metformin	hBMSC	Ameliorating disc cell senescence *in vitro* and optimizing the potential for the treatment of IDD.	Metformin-induced AMPK activation induces the phosphorylation of SNAP29, which in turn mediates the transfer of ITIHT4 to MVBs, leading to the accumulation of ITIH4 in the released exosomes.	[97]
Glycyrrhetinic acid (GA)	AD-MSC	Promoting therapeutic effect against acute liver ischemia-reperfusion injury.	/	[98]
Kartogenin	BMSCs	Promoting the effect on cartilage regeneration in a rat OA model.	Reduction of the expression of COLI in chondrocytes.	[99]
Thrombin	HUCB-MSC	Boosting the biogenesis of MSC-derived EVs and enriching their cargo contents.	These effects are achieved *via* PAR-1-mediated pathways and partly *via* the PAR-1-independent, PAR-3-mediated activation of Rab5, EEA-1, and the ERK1/2 and AKT signaling pathways.	[100]
Oridonin	BMSC	Improving the therapeutic potential against ischemia-reperfusion injury in rats.	These effects are achieved through participating in the autophagic process of cardiomyocytes	[101]
Heme oxygenase-1 (HO-1)	hBMSC	Improving the efficacy of exosomes to alleviate myocardial infarction (MI)	Expression of miR-183-5p in exosomes is elevated and then inhibiting cardiomyocyte senescence through regulation of HMGB1/ERK pathway	[102]
Atorvastatin (ATV)	BMSC	Enhancing biological functions of endothelial cells in the treatment of diabetic skin defects.	These effects are achieved *via* the AKT/eNOS pathway by upregulating miR-221-3p.	[103]
Atorvastatin (ATV)	BMSC	Exhibiting more potent cardioprotective function in a rat model of AMI.	These effects are achieved through the upregulation of long noncoding RNA H19.	[104]
Melatonin	BMSC	Promoting microglia to M2-like polarization and alleviating spinal cord injury.	Ubiquitin-specific protease 29 (USP29) increases markedly in exosomes and stabilizes nuclear factor-like 2 (NRF2).	[105]
Melatonin	BMSC	Improving functional recovery and vessel repair in a murine hindlimb ischemia model with CKD.	Increasing the expression of cellular prion protein [PrP (c)] in exosomes.	[106]
Melatonin	hBMSC	Promoting diabetic wound healing.	Activating the PTEN/AKT signaling pathway.	[107]
Melatonin	BMSC	Improving the therapeutic potential against renal ischemia-reperfusion injury in rats.	/	[108]
Blue light	HUC-MSC	Promoting proangiogenic ability in murine matrigel plug and skin wound models.	Upregulation of miR-135b-5p and miR-499a-3p in MSC-exosomes.	[112]
Extrusion	AD-MSC	Improving robust bone regeneration effects in mouse nonhealing calvarial defects.	Inhibition of miR-29a in MSC-exosomes.	[113]
Bioglass	hBMSC	Promoting vascularization of endothelial cells and facilitating intradermal angiogenesis.	Decreasing microRNA-342-5p, while increasing microRNA-1290 in MSC-exosomes.	[114]
Titanium surfaces	hBMSC	Inducing elevated secretion of exosomes and enhancing angiogenesis in vitro	Increasing the expression of angiogenesis-related factors in exosomes	[109]
Low-intensity pulsed ultrasound (LIPUS)	BMSC	Enhancing the effect of exosomes on cartilage regeneration in osteoarthritis	These benefits are achieved by inhibiting the activation of the nuclear factor-*κ*B (NF-*κ*B) pathway	[110]
PG/TCP (PEGMC with *β*-TCP)	BMSC	Promoting osteogenesis and spinal fusion	/	[111]
